# Severe COVID-19 and chronic kidney disease: bidirectional mendelian randomization study

**DOI:** 10.1186/s12985-023-02280-z

**Published:** 2024-01-29

**Authors:** Haishan Lin, Bangwei Cao

**Affiliations:** Cancer Centre, Beijing Friendship Hospital, Capital Medical University, 95 Yong An Road, Xicheng District, Beijing, 100050 China

**Keywords:** Bidirectional two-sample mendelian randomization, COVID‐19, Chronic Kidney Disease

## Abstract

Traditional observational research has revealed an association between severe COVID-19 and chronic kidney disease (CKD). It is unclear whether there is a causative connection between them. Our goal was to determine whether genetically predicted CKD is associated with the risk of critical COVID-19. We aimed to investigate potential underlying genetic mechanisms that could explain this relationship, paving the way for personalized risk assessment and targeted interventions to mitigate the effects of COVID-19 on individuals with CKD. Using combined data from a GWAS on European ancestry and CKD (n = 117,165) and critical COVID-19 (n = 1,059,456), bidirectional Mendelian randomization analysis was performed. Four single nucleotide polymorphisms (SNPs) were chosen from the genome as CKD instrumental variables (IVs). In addition to MR‒Egger regression, weighted mode approaches, and weighted medians, we employed the inverse-variance weighted estimate as our primary analytical method. A significant association of CKD with critical COVID-19 (OR = 1.28, 95% confidence interval [CI]: 1.04–1.58, *p* = 0.01811) was found. However, using 6 genome-wide significant SNPs as IVs for critical COVID‐19, we could not discover a meaningful correlation between severe COVID‐19 and CKD (OR = 1.03, 95% CI: 0.96–1.10, *p* = 0.3947). We found evidence to support a causal relationship between CKD and severe COVID-19 in European population. This underscores the need for comprehensive monitoring and specialized care strategies for individuals with CKD to mitigate the heightened risk and severity of COVID-19 complications.

**Supplementary Information:**

The online version contains supplementary material available at 10.1186/s12985-023-02280-z.

## Introduction

The coronavirus disease 2019 (COVID-19) pandemic, caused by SARS-CoV-2, has brought considerable attention to the association between COVID-19 and renal impairment [1]. Early in the pandemic, studies highlighted the alarming incidence of acute kidney injury (AKI) in severe COVID-19 cases, shedding light on the impact of the virus on renal function [2]. Understanding the intricacies of this relationship is crucial for effective management and targeted interventions for individuals with COVID-19 and renal complications. According to global reports, SARS and COVID-19 deaths were more common among those with chronic kidney disease (CKD) and those who needed dialysis or a kidney transplant [3]. Early studies, primarily from China, found that mortality and severe COVID-19 were both influenced by an underlying CKD [4]. In addition, CKD was identified as a mortality risk factor among COVID-19 patients in a large-scale UK analysis comprising 17 million cases, with organ transplantation and a glomerular filtration rate (GFR) lower than 30 mL/min/1.73 m2 being associated with a high risk [5]. Patients receiving in-center dialysis were identified to be more susceptible to contracting SARS-CoV-2 [6]. Male sex, underlying CKD, heart failure, age, and a BMI greater than 40 kg/m2 were discovered to be powerful predictors of severe illness and hospital admission in a New York study among more than 5,000 people with COVID-19 [7]. The seroprevalence of SARS-CoV-2 antibodies in in-center dialysis patients varied from 3.5 to 27.2%, according to a national study that compiled data from approximately 1,300 dialysis centers in the USA. In some areas of the country, the prevalence was more than 10% the national average among all people [8].

Building upon this evidence, we speculate that there is a certain correlation between severe COVID-19 and CKD. However, it is not clear whether CKD directly causes severe COVID-19, or CKD is a confounding factor, and whether CKD and severe COVID-19 are causal inversion. Our study aimed to further investigate the impact of genetically predicted CKD on the risk and severity of critical COVID-19, providing crucial insights for targeted interventions and patient care.Since any detected relationships could be the result of confounding factors, it is challenging to draw any conclusions about causality from observational studies. To identify genetic proxies for CKD and severe COVID-19 risk, we used data from large-scale genetic association studies. We then applied these genetic proxies to Mendelian randomization (MR) analysis to establish a connection between the causes of the two conditions while reducing confounding factor bias and reverse causality. Through this rigorous approach, we aimed to provide stronger evidence and deeper insights into the possible causal link between CKD and COVID-19, contributing to a better understanding of their interplay.

## Materials and methods

### Study design

**Fig. 1:**
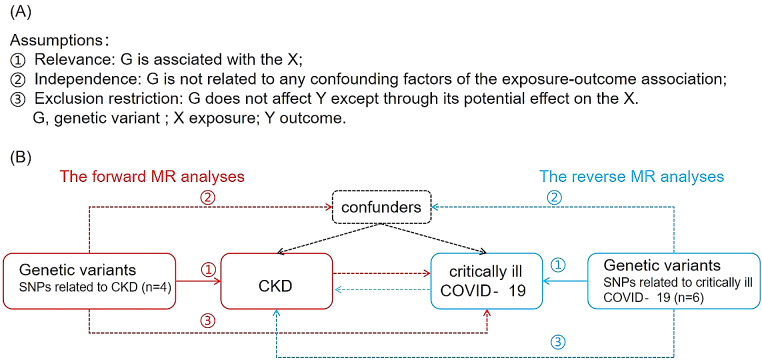
Description of the study design of the bidirectional MR analysis. (**A**) The three central assumptions of MR analysis. (**B**) The forward MR analyses are depicted in red, with CKD serving as the exposure factor and severe COVID-19 as the outcome. The reverse MR analyses are shown in blue, with severe COVID-19 as the exposure factor and CKD as the outcome. LD, linkage disequilibrium; CKD, chronic kidney disease; MR, Mendelian randomization; SNPs, single nucleotide polymorphisms

A basic overview of this bidirectional MR study of severe CKD and COVID-19 is shown in Fig. 1. GWAS of CKD were comprised by 43 studies, for a total sample size of 117,165, including 12,385 CKD cases. Supplementary Table 1 provides the details of all study cohorts. GWAS of sever COIVD19 were comprised by 13 studies of European ancestry, for a total sample size of 1,054,664, including 4,792 sever COIVD19. Supplementary Table 2 provides the details of all study cohorts. By using summary statistics from GWASs, we conducted two MR analyses to examine the connection between CKD and severe COVID-19. In contrast to forward MR studies that used CKD as the exposure factor and COVID-19 as the outcome, this MR analysis employed severe COVID-19 as the exposure factor and CKD as the outcome. This unique perspective provides valuable insights into the potential bidirectional relationship between these conditions, contributing to a comprehensive understanding of their interplay and informing future interventions. The primary MR theories are shown in Fig. 1. Since this study was based on publicly available summary statistics, no ethical approval was required.

### Selection of IVs for MR analyses

Two distinct pooled GWAS datasets were used to choose the IVs that would work best for the MR analysis. First, SNPs that met the criterion for genome-wide significance (*p* < 5 × 10 − 8) were chosen. Second, linkage disequilibrium LD was used to shortlist the appropriate SNPs, minimum LD Rsq value set as 0.8, and as determined by r 2 > 0.001 in the European 10,000 Genome reference panel. The results of SNPs with *p* < 5 × 10 − 8 were then excluded. Palindromic SNPs with intermediate allele frequencies were excluded when exposure and outcome data were combined, and MAF threshold for aligning palindromes 0.3. This stringent approach ensured the reliability and accuracy of the analysis, allowing for a more precise assessment of the relationship between the variables of interest and minimizing potential confounding factors.

### Data sources and IV selection for CKD

Forty-three European cohorts were used in our consolidated meta-analysis for CKD, which were obtained from the CKDgen (CKDGEN Meta-Analysis datasets [uni-freiburg.de]), consisting of 12,385 cases and 104,780 controls (Supporting Information, Table 1) [9]. Four independent genetic CKD IVs were obtained according to the following 3 criteria: (1) *p* < 5 × 10^− 8^ was the genome-wide significance threshold; (2) r^2^ < 0.001, suggesting no linkage disequilibrium among the SNPs; and (3) no influence on other potential risk factors. Detailed information on the IVs is presented in the Supporting Information, Table 3.

### Data sources and IV selection for severe COVID-19

The COVID-19 Host Genetics Initiative (https://www.covid19hg.org/results/r5/) provided us with unified meta-analysis data, which included 4,792 cases and 1,054,664 controls from 14 European cohorts of patients with severe COVID-19 (Supporting Information, Table 2) [10] The patients with severe COVID-19 included in our study were hospitalized either due to their clinical symptoms resembling laboratory-confirmed SARS-CoV-2 infection and requiring respiratory assistance or because they had unfortunately succumbed primarily to COVID-19-related complications. These patient selection criteria ensured a representative cohort for investigating the impact and outcomes of severe COVID-19 cases, facilitating a comprehensive analysis of the disease severity and associated risks. If pertinent information was available, controls were individuals with matching genetic ancestry but no confirmed SARS-CoV-2 infection. In this GWAS, seven SNPs related to patients with severe COVID-19 were found, and they were chosen as IVs. To perform reverse MR analysis, we selected the proper IVs. One SNP with *p* > 5 × 10^− 8^ was eliminated out of a total of 7 SNPs. Thus, in the reverse MR analysis, we ultimately included 6 SNPs as IVs (Supporting Information, Table 4).

### Statistical analysis

To calculate probable causal relationships between severe COVID-19 and CKD, we employed the random-effects inverse-variance weighted (IVW) technique. This widely recognized statistical approach enabled us to account for potential heterogeneity and provide robust estimates of the causal effects. The weighted median estimator model (WME), weighted model-based method (WM), and MR-Egger regression model (MER) were also used to estimate causal effects [11–13, 15]. The heterogeneity between IVs was tested by Cochrane’s Q-statistic. The leave-one-out sensitivity test was used to judge the stability of the MR results by excluding IVs one by one [16]. Directional pleiotropy was checked and corrected based on the intercept obtained from the MER analysis and the MR pleiotropy residual sum and outlier test (MR-PRESSO) [17]. This two-sample MR analysis was performed using R software (version 4.2.0) with TwoSampleMR (version 0.5.6) and MR-PRESSO packages (version 1.0.0).

## Results

### The causal effect of CKD on severe COVID-19

**Fig. 2:**
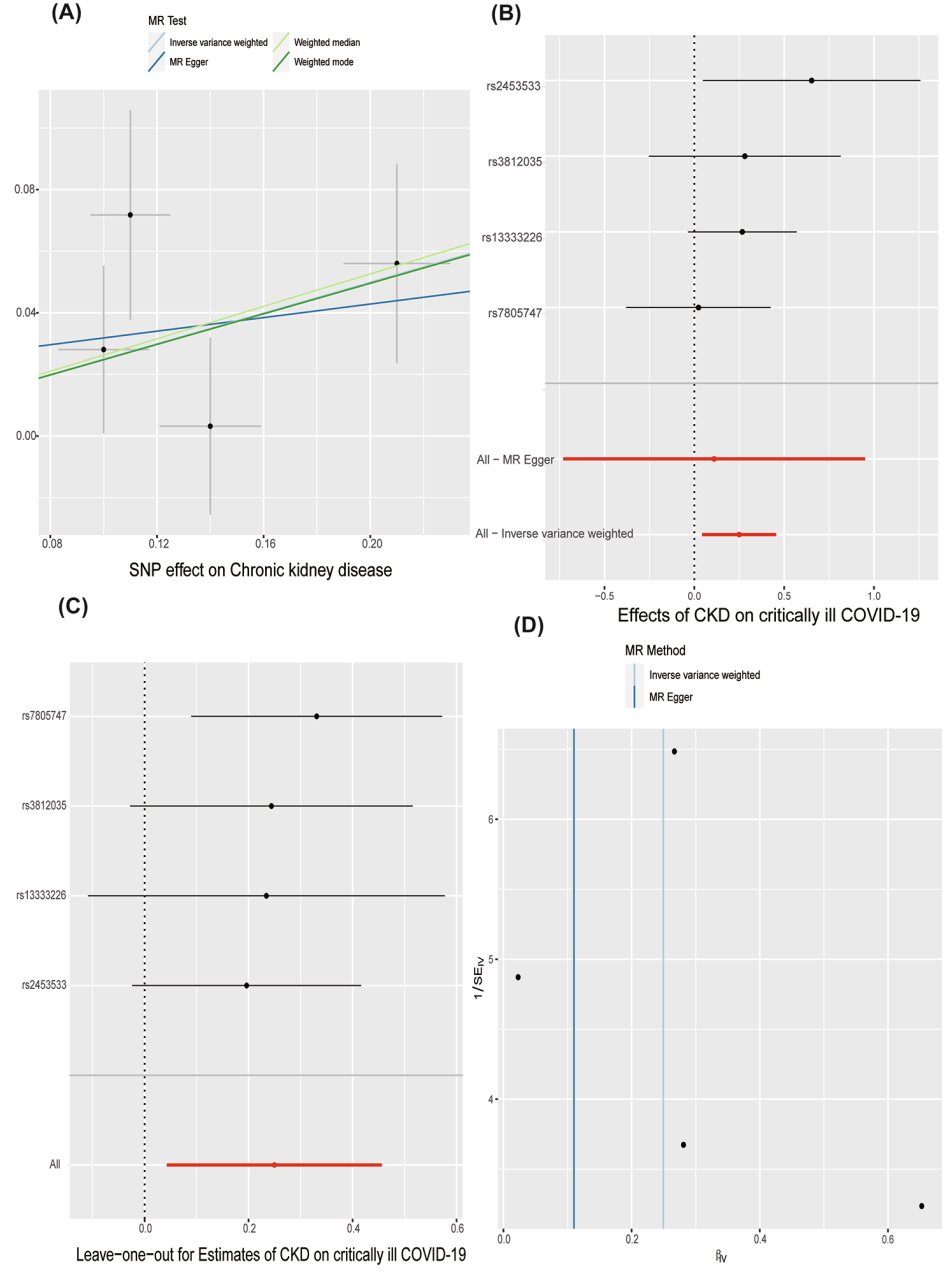
Forward MR analyses: Causal effect of CKD on severe COVID-19. **(A)** Scatter plot illustrating the relationship between severe COVID-19 and CKD using all four approaches employed in this investigation. **(B)** A forest plot with the MR estimate and 95% CI values for the SNPs (black line), as well as the MR-Egger and IVW findings, is displayed. **(C)** To determine if any specific IVs were mediating the causal impact, leave-one-out analyses were conducted. **(D)** To find any significant heterogeneity in the observed associations, a funnel plot was utilized. CI, confidence interval; IVW, inverse-variance weighted; CKD, chronic kidney disease; MR, Mendelian randomization; SNPs, single nucleotide polymorphisms

The characteristics of the four SNPs employed as IVs for CDK are presented in Supplementary Tables S3, along with the scatter plot in Fig. 2A. In forward study, CKD as expose and sever COVID-19 as outcome, there was no evidence of heterogeneity in the Cochran’s Q test in the MR‒Egger and IVW techniques (Table 2, all Cochran’s Q *p* values > 0.001) and Fig. 2D (funnel plot), and hence a fixed-effects model was adopted in the forwad MR analysis. The IVW outcome revealed that genetically predicted critical CKD was substantially linked with severe COVID-19 (OR and 95% CI: 1.28, 1.04–1.58, *p* = 0.018), as reported in Table 1 and the scatter plot presented in Fig. 2B, indicating that CKD was associated with a 28.3% higher risk of critical COVID-19. The results using the weighted median suggested that genetically predicted critical CKD was significantly associated with critical COVID-19 (OR and 95% CI: 1.30, 1.01–1.67; *p* = 0.035) (Table 1). Among other models, no significant association was found. No outlier between CKD and sever COVID-19 was identified by the MR-PRESSO test, and the robustness of results was confirmed by the leave-one-out sensitivity test Fig. 2C.

**Table 1:** CKD and its association with sever COVID-19 in the MR analyses

Exposure	Outcome	No. of SNPs	Methods	OR (95% CI)	β (SE)	*p*
Forward MR analyses						
CKD	sever COVID-19	4	IVW	1.28(1.04,1.58)	0.25(0.11)	0.018
			MR-Egger	1.12(0.48,2.59)	0.11(0.43)	0.822
			Weighted median	1.30(1.01,1.67)	0.26(0.13)	0.038
			Weighted mode	1.28(0.88,1.88)	0.25(0.19)	0.286
Reverse MR analyses						
sever COVID-19	CKD	6	IVW	1.03(0.96,1.10)	0.03(0.03)	0.395
			MR-Egger	1.03(0.89,1.23)	0.03(0.09)	0.737
			Weighted median	1.05(0.98,1.13)	0.05(0.04)	0.2
			Weighted mode	1.05(0.97,1.14)	0.05(0.04)	0.25

### The causal effect of severe COVID-19 on CKD

**Fig. 3:**
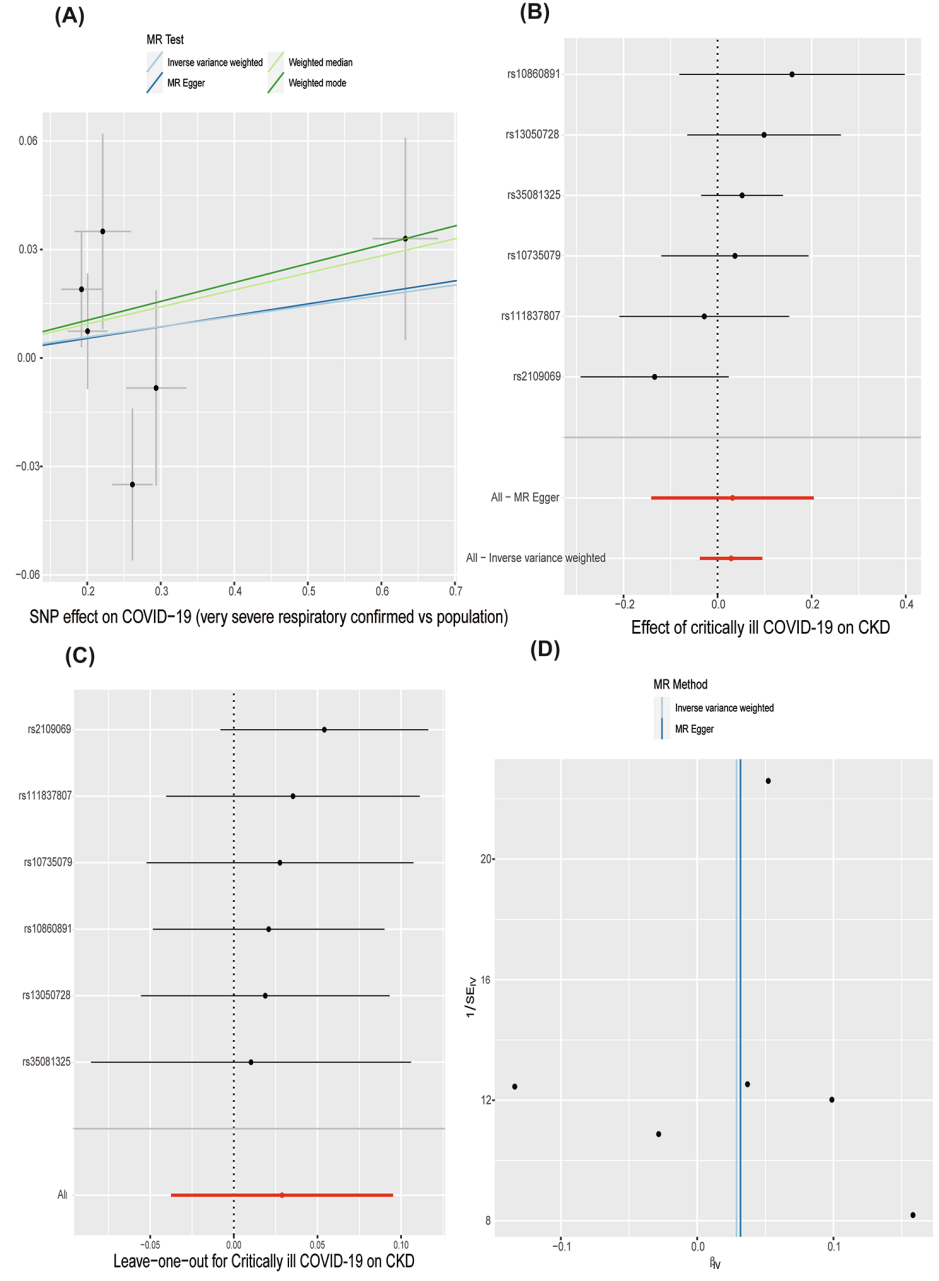
Reverse MR analyses: Causal effect of CKD on severe COVID-19. **(A)** Scatter plot demonstrating the relationship between severe COVID-19 and CKD using all four techniques employed in this investigation. **(B)** The MR estimate and 95% CI values for the SNPs are displayed in a forest plot, along with the MR‒Egger and IVW findings at the bottom. **(C)** To determine whether any particular IV was mediating the causal impact, leave-one-out analyses were carried out. **(D)** Any substantial heterogeneity in the observed associations was found using a funnel plot. Cl, confidence interval; IVW, inverse-variance weighted; CKD, chronic kidney disease; MR, Mendelian randomization; SNPs, single nucleotide polymorphisms

The characteristics of the six SNPs employed as IVs for sever COIVD-19 are presented in Supplementary Tables S4, along with the scatter plot in Fig. 3A. In reverse study, sever COVID-19 as expose and CKD as outcome, there was no evidence of heterogeneity in the Cochran’s Q test in the MR‒Egger and IVW techniques (Table 2, all Cochran’s Q *p* values > 0.001) and Fig. 2D (funnel plot), and hence a fixed-effects model was adopted in the reverse MR analysis. The scatter plot (Fig. 3A) and forest plots (Fig. 3B), as shown in Table 1, revealed that severe COVID-19 was not causally related to CKD (OR and 95% CI: 1.03, 0.96–1.10; *p* = 0.395). The remaining three findings mirrored those of the IVW method (Table 1). The MR-PRESSO (Table 2, global test *p* = 0.338) and MR-Egger regression analysis (Table 2, intercept =-0.00094, *p* = 0.972) results indicated that there was no possible pleiotropy. Additionally, leave-one-out analyses showed that the results were stable.

**Table 2:** Pleiotropy and heterogeneity analyses

Exposure	Outcome	No. of SNPs	MR-Egger regression		MR-PRESSO		Heterogeneity analyses		
			Intercept	p_intercept	Global test p	Correct *p* *	Method	Q	Q_pval
CKD	sever COVID-19	4	0.021	0.766	4.498037	0.532	IVW	2.948	0.3998
							MR Egger	2.787	2.787
sever COVID-19	CKD	6	-0.00094	0.972	9.058734	0.338	IVW	6.608	0.2514
							MR Egger	6.606	0.1582

Abbreviations: IVW, inverse-variance weighted; MR-PRESSO, Mendelian randomization pleiotropy residual sum and outlier; CKD, chronic kidney disease; SNPs, single nucleotide polymorphisms. *Correct p is computed by eliminating instrument variations with horizontal pleiotropy if pleiotropy is detected by the MR-PRESSO global test and there is a sizable difference between the outcomes before and after the outlier is eliminated

## Discussion

Genetically proxied CKD was linked to a higher probability of COVID-19 respiratory failure or severe COVID-19, according to this bidirectional two-sample MR study. However, it was not possible to produce any conclusive evidence linking the risk of CKD to genetically proxied severe COVID-19.

As already indicated, numerous previous observational studies have shown that CKD is probably linked to critical COVID-19. Our results provide credence to the idea that CKD increases a person’s vulnerability to severe COVID-19. Most patients with CKD have a long-term usage of hormones and immunosuppressants, which can further aggravate the injury of COVID-19 infection [18]. Excluded the condition of these confounding factors, MR study indicates that CDK is still related to severe COVID-19.This causal association may be explained by a number of different processes, particularly immunological dysregulation of CDK patients, such as loss of immunoglobulin from urine, and insufficient complement function, which exacerbates the course of COVID − 19 infection [19]. Furthermore, renal edema of CDK may cause pulmonary edema and further exacerbate the ensuing respiratory symptoms.

Previous research also recognized AKI as a major complication of severe COVID-19 [20], in addition to the discovery of CKD as a risk factor for poor outcomes in patients with COVID-19. A previous investigation revealed that SARS-CoV-2 directly infected kidney cells, causing cell damage that led to fibrosis [21]. Furthermore, individuals with acute SARS-CoV-2 infection frequently have kidney involvement, and subclinical inflammation and injury may last for several months, causing a progressive deterioration in kidney function that eventually results in CKD [22]. However, our findings did not support the idea that severe COVID-19 causes CKD on its own. The inability of genetically proxied severe COVID-19 to accurately predict long COVID-19 may be to blame for this outcome. Additionally, the relationship between COVID-19 and CKD is complex. In addition to direct renal toxicity, SARS-CoV-2 infection may cause hemodynamic alterations and cytokine release, which affect the functions of the kidneys [23–24]. As a result, it appears that there is no direct causal relationship between COVID-19 and CKD.

The greatest advantage of the current investigation over conventional observational studies is that reverse causality and confounding bias were avoided in the causal estimate derived using MR. Additionally, using complete GWAS data for MR analysis can increase the precision of the projected effect. However, this study has several limitations. First, it was impossible to completely eliminate evident heterogeneity using Cochran’s Q in the forward or reverse MR analysis. Furthermore, because the study sample was primarily European, the findings cannot be extrapolated to people of other races or nationalities. And there are still a large number of observational research data showing that there is an association between CKD and COVID-19 in non-European population [7–8]. In the future, the deep relationship between CKD and COVID-19 can be further explored.

We investigated the relationship between severe COVID-19 and CKD using large-scale genetic summary data. Our results confirmed genetically predicted CKD and the likelihood of severe COVID-19 in European population. These findings provide robust evidence supporting the notion that CKD plays a major role in the severity of COVID-19 outcomes, emphasizing the critical need for targeted interventions and specialized care tailored to individuals with underlying kidney disease.

### Electronic supplementary material

Below is the link to the electronic supplementary material.

Supplementary Material 1

## Data Availability

The data that support the findings of this study are available in the COVID-19 Host Genetics Initiative and CKDgen. These data were derived from these resources available in the public domain.

## Declarations

### Patient and public involvement

Patients or the public were not involved in the design, conduct, reporting, or dissemination plans of our research. The data came from public databases.
