# Supplementary material for: Severe COVID-19 and chronic kidney disease: bidirectional mendelian randomization study
Source: Virol J. 2024 Jan 29;21:32. doi: 10.1186/s12985-023-02280-z (PMC10823696; doi:10.1186/s12985-023-02280-z)
Supplement: Supplementary file 1 — Supplementary Material 1 [file 12985_2023_2280_MOESM1_ESM.docx]

| **Supplemetary Table 1.** Details of CKD data sources used in this study. | | | | | | | | | | |
| --- | --- | --- | --- | --- | --- | --- | --- | --- | --- | --- |
| **Study Name** | **Sample size** | | | **Women**  **% (N)** | **Age**  **Mean**  **(SD)** | **eGFRcrea**  **Mean(SD)**  **ml / min /**  **1.73m2** | **eGFRcys**  **Mean(SD)**  **ml / min /**  **1.73m2** | **CKD**  **% (N)** | **HTN**  **% (N)** | **DM**  **% (N)** |
|  | **eGFRcrea** | **CKD** | **eGFRcys** |  |  |  |  |  |  |  |
| **Discovery studies** | | | | | | | | | | |
| **Advance** | 2287 | 2287 | NA | 32.8  (755) | 67(7) | 85.1(29.2) | NA | 14.7  (337) | 47.6  (1096) | 100.0  (2287) |
| **AGES** | 3219 | 3219 | NA | 58.0  (1867) | 76(5) | 73.0(20.0) | NA | 24.2  (781) | 80.6  (2595) | 11.5  (368) |
| **ASPS** | 848 | 848 | NA | 56.8  (482) | 65(8) | 96.5(39.9) | NA | 8.1  (69) | 72.5  (615) | 9.2  (78) |
| **AUSTWIN** | 9592 | 3320 | NA | 60.9  (5846) | 46(13) | 98.3(26.2) | NA | 10.5  (350) | NA | NA |
| **BLSA** | 723 | 723 | NA | 46.1  (333) | 70(15) | 80.3(23.1) | NA | 17.4  (126) | 21.9  (147) | 7.7  (55) |
| **BMES** | 2437 | 2437 | NA | 56.8  (1385) | 69(9) | 78.7(20.2) | NA | 13.2  (322) | 76.4  (1861) | 10.9  (265) |
| **CROATIA- KORCULA** | 888 | 888 | NA | 64.0  (568) | 56(14) | 87.3(20.6) | NA | 7.5  (67) | 54.2  (474) | 13.1  (116) |
| **CROATIA-**  **SPLIT** | 478 | 478 | NA | 59.8  (286) | 49(15) | 104.8(23.8) | NA | 5.0  (24) | 39.4  (186) | 5.0  (24) |
| **CROATIA-VIS** | 768 | 768 | NA | 58.6  (450) | 57(15) | 88.2(22.1) | NA | 6.9  (53) | 52.2  (396) | 12.0  (91) |
| **EGCUT Omni** | 261 | 261 | NA | 73.4  (193) | 81(9) | 71.2(24.1) | NA | 34.2  (90) | 82.5  (217) | 21.8  (57) |
| **ERF** | 2561 | 2561 | NA | 55.3  (1416) | 49(14) | 93.3(21.4) | NA | 3.7  (96) | 52.9  (1355) | 6.1  (155) |
| **GENOA** | 1163 | 1163 | NA | 56.3  (655) | 59(10) | 87.7(24.0) | NA | 10.7  (125) | 73.3  (852) | 15.3  (178) |
| **HCS** | 1235 | 1235 | NA | 49.6  (630) | 66(7) | 79.9(18.1) | NA | 11.3  (144) | 45  (544) | 10.5  (127) |
| **HPFS** | 818 | 818 | NA | 0.0  (0) | 65(8) | 85.2(22.7) | NA | 9.5  (78) | 59  (479) | 100.0  (818) |
| **HYPERGENES**  **HTN cases** | 1591 | 1591 | NA | 33.0  (525) | 48(10) | 94.5(23.1) | NA | 3.7  (59) | 100.0  (1591) | 0.0  (0) |
| **HYPERGENES**  **HTN ctrls** | 1662 | 1662 | NA | 39.6  (659) | 60(10) | 87.8(19.0) | NA | 4.9  (81) | 0.0  (0) | 0.0  (0) |
| **INGI-CILENTO** | 821 | 821 | NA | 54.9  (451) | 54(18) | 88.7(21.8) | NA | 8.0  (66) | 38.4  (315) | 10.5  (86) |
| **INGI-FVG** | 874 | 874 | NA | 59.4  (519) | 52(16) | 90.6(21.8) | NA | 6.0  (52) | 48.8  (427) | 6.7  (59) |
| **INGI-VAL**  **BORBERA** | 1636 | 1636 | NA | 55.8  (913) | 55(18) | 89.2(23.3) | NA | 8.5  (139) | 43.8  (717) | 6.5  (107) |
| **JUPITER** | 8780 | 8780 | NA | 32.2  (2827) | 66(8) | 80.1(18.1) | NA | 11.5  (1008) | 55.8  (4901) | 0.4  (37) |
| **NHS** | 786 | 786 | NA | 100.0  (786) | 59(6) | 86.2(22.1) | NA | 10.7  (84) | 70  (554) | 100.0  (786) |
| **NSPHS** | 565 | 565 | NA | 53.1  (300) | 52(18) | 91.0(22.1) | NA | 5.7  (32) | 43.4  (242) | 7.8  (44) |
| **OGP-TALANA** | 862 | 862 | NA | 57.3  (494) | 51(19) | 91.2(23.6) | NA | 7.5  (65) | 37.3  (322) | 5.1  (44) |
| **ORCADES** | 704 | 704 | NA | 53.6  (377) | 54(15) | 89.4(20.7) | NA | 6.8  (48) | 41.8  (287) | 4.0  (28) |
| **POPGEN** | 1163 | 1163 | NA | 44.4  (516) | 55(14) | 88.1(18.8) | NA | 5.1  (59) | 46.8  (541) | 3.8  (44) |
| **PROSPER-**  **PHASE** | 5236 | 5236 | NA | 51.7  (2718) | 75(3) | 72.0(21.4) | NA | 29.6  (1549) | 62.1  (3251) | 10.4  (544) |
| **RS-I** | 4390 | 4390 | NA | 61.4  (2696) | 70(9) | 77.1(17.2) | NA | 13.7  (600) | 34.1  (1497) | 10.7  (470) |
| **RS-II** | 1863 | 1863 | NA | 54.5  (1015) | 65(8) | 81.3(17.2) | NA | 9.1  (169) | 28.4  (530) | 11.1  (207) |
| **SAPALDIA** | 1444 | 1444 | NA | 51.0  (737) | 52(11) | 90.3(17.3) | NA | 3.1  (44) | 27.4  (389) | 2.8  (40) |
| **SORBS** | 856 | 856 | NA | 58.5  (501) | 49(16) | 92.2(19.0) | NA | 4.1  (35) | 53.2  (455) | 9.3  (80) |
| **WGHS** | 21940 | 23186 | NA | 100.0  (21,940) | 55(7) | 90.0(22.5) | NA | 6.1  (1329) | 24.5  (5374) | 2.5  (554) |
| **Bus Santé** | 4408 | 4408 | NA | 49.4  (2178) | 58(11) | 85.6(15.6) | NA | 4.3  (186) | 28.3  (1249) | 7.4  (327) |
| **ESTHER** | 3604 | 3604 | NA | 55.6  (2004) | 62(7) | 90.3(34.4) | NA | 15.7  (565) | 57.5  (2073) | 15.9  (2073) |
| **GHS 1** | 2995 | 2995 | NA | 48.5  (1452) | 56(11) | 87.3(16.5) | NA | 3.7  (112) | 52.6  (1575) | 7.2  (215) |
| **GHS 2** | 1179 | 1179 | NA | 50.0  (1147) | 55(11) | 86.7(16.2) | NA | 4.5  (53) | 48.4  (570) | 7.6  (90) |
| **GSK** | 1721 | 1721 | NA | 66.6  (1147) | 51(13) | 92.3(22.6) | NA | 5.5  (95) | 43.7  (752) | 4.6  (80) |
| **IPM_EA_Illu** | 1307 | 1307 | NA | 48.6  (635) | 68(9) | 86.1(27.8) | NA | 14.8  (194) | 55.1  (720) | 15.0  (185) |
| **OGP** | 9554 | 5884 | NA | 56.9  (5440) | 50(17) | 98.6(35.0) | NA | 8.1  (776) | 36.0  (3443) | 6.2  (596) |
| **Vanderbilt**  **Omni1** | 3221 | 3221 | NA | 47.3  (1525) | 54(19) | 80.0(36.9) | NA | 27.7  (891) | 70.5  (2271) | 18.0  (581) |
| **Vanderbilt**  **Omni5** | 1129 | 1129 | NA | 46.9  (529) | 50(21) | 89.0(44.4) | NA | 21.7  (245) | 58.2  (657) | 33.3  (376) |
| **Vanderbilt**  **660W** | 2299 | 2299 | NA | 56.5  (1298) | 56(17) | 78.6(23.9) | NA | 20.6  (474) | 57.2  (1316) | 17.9  (411) |
| **ARIC** | 8982 | 8982 | 7145 | 53.1  (4767) | 62(6) | 81.4(17.5) | 84.2(19.7) | 8.7  (782) | 40.7  (3643) | 14.2  (1276) |
| **LURIC** | 3056 | 3056 | 3054 | 30.0  (917) | 63(11) | 86.0(21.7) | 84.7(22.6) | 10.0  (305) | 72.7  (2223) | 32.6  (996) |

| **Supplemetary Table 2.** Details of critically ill COVID‐19 data sources used in this study. | | | | | | | | |
| --- | --- | --- | --- | --- | --- | --- | --- | --- |
| **Factors** | **Study Name** | **Site Abbreviation** | **Population** | **Cases (average age)** | **Controls (average age)** | **No. of SNPs** | **Sample size** | **Source** |
| Very severe respiratory confirmed covid vs. population | Biobanque Quebec COVID-19 | BQC19 | European | 88 (72.29 years) | 552 (67.55 years) | 6 | 1,054,664 | https://www.covid19hg.org/results/r5/ |
|  | BelCovid | BelCovid | European | 182 (67 years) | 1,477 (56 years) |  |  |  |
|  | Columbia University COVID-19 Biobank | CU | European | 203 (63 years) | 2,149 (unknown) |  |  |  |
|  | Finngen | FinnGen | European | 68 (51.8 years) | 238,643 (58.6 years) |  |  |  |
|  | GEN-COVID,reCOVID | GENCOVID | European | 724 (63.29 years) | 2,443 (48.15 years) |  |  |  |
|  | MyCode Health Initiative | GHS_Freeze_145 | European | 53 (56.8 years) | 112,862 (56.8 years) |  |  |  |
|  | The genetic predisposition to severe COVID-19 | SweCovid | European | 77 (60.4 years) | 3,748 (60.4 years) |  |  |  |
|  | idipaz24genetics | idipaz24genetics | European | 59 (62.29 years) | 75 (44.05 years) |  |  |  |
|  | Amsterdam UMC COVID study group | Amsterdam_UMC_COVID_study_group | European | 66 (66.56 years) | 1,413 (53.38 years) |  |  |  |
|  | Determining the Molecular Pathways and Genetic Predisposition of the Acute inflammatory Process Caused by SARS-CoV-2 | SPGRX | European | 101 (69.32 years) | 302 (47.92 years) |  |  |  |
|  | GenOMICC (Genetics Of Mortality in Critical Care) | genomicc | European | 1,676 (59.05 years) | 8,380 (67.98 years) |  |  |  |
|  | COVID19-Hostage | Italy_HOSTAGE | European | 698 (59.05 years) | 1,255 (67.98 years) |  |  |  |
|  | COVID19-Hostage | Spain_HOSTAGE | European | 302 (59.05 years) | 925 (67.98 years) |  |  |  |
|  | 23andMe | 23ANDME | European | 495 (51 years) | 680,440 (51 years) |  |  |  |

| **Supplementary Table 3.** Summary of genetic variants (n=4) used to estimate the effect of CKD on critically ill COVID-19 in MR analyses. | | | | | | | |
| --- | --- | --- | --- | --- | --- | --- | --- |
| **rsid** | **Chr** | **Pos** | **Effect allele** | **Other allele** | **P-value** | **Beta** | **Se** |
| rs13333226 | 16 | 20365654 | G | A | 3.80014e-26 | -0.21 | 0.020 |
| rs2453533 | 15 | 45641225 | A | C | 5.40008e-12 | 0.11 | 0.015 |
| rs7805747 | 7 | 151407801 | A | G | 4.27388E-09 | 0.14 | 0.019 |
| rs3812035 | 5 | 176817143 | T | G | 5.85651E-20 | 0.10 | 0.017 |

| **Supplementary Table 4.** Summary of genetic variants (n=6) used to estimate the effect of critically ill COVID-19 on CKD in MR analyses. | | | | | | | |
| --- | --- | --- | --- | --- | --- | --- | --- |
| **rsid** | **Chr** | **Pos** | **Effect allele** | **Other allele** | **P-value** | **Beta** | **Se** |
| rs10735079 | 12 | 113380008 | A | G | 1.55310e-13 | 0.20 | 0.027 |
| rs10860891 | 12 | 103014757 | A | C | 7.85199e-09 | -0.22 | 0.038 |
| rs111837807 | 6 | 31121232 | C | T | 7.92866e-13 | 0.29 | 0.040 |
| rs13050728 | 21 | 34615210 | C | T | 2.30622e-12 | -0.19 | 0.027 |
| rs2109069 | 19 | 4719443 | A | G | 1.46386e-21 | 0.26 | 0.027 |
| rs35081325 | 3 | 45889921 | T | A | 5.28567e-46 | 0.63 | 0.044 |

| **Supplementary Table S3.** Summary of genetic variants (n=4) used to estimate the effect of CKD on sever COVID-19 in MR analyses. | | | | | | | |
| --- | --- | --- | --- | --- | --- | --- | --- |
| **rsid** | **Chr** | **Pos** | **Effect allele** | **Other allele** | **P-value** | **Beta** | **Se** |
| rs13333226 | 16 | 20365654 | G | A | 3.80014e-26 | -0.21 | 0.020 |
| rs2453533 | 15 | 45641225 | A | C | 5.40008e-12 | 0.11 | 0.015 |
| rs7805747 | 7 | 151407801 | A | G | 4.27388E-09 | 0.14 | 0.019 |
| rs3812035 | 5 | 176817143 | T | G | 5.85651E-20 | 0.10 | 0.017 |

| **Supplementary Table S4.** Summary of genetic variants (n=6) used to estimate the effect of sever COVID-19 on CKD in MR analyses. | | | | | | | |
| --- | --- | --- | --- | --- | --- | --- | --- |
| **rsid** | **Chr** | **Pos** | **Effect allele** | **Other allele** | **P-value** | **Beta** | **Se** |
| rs10735079 | 12 | 113380008 | A | G | 1.55310e-13 | 0.20 | 0.027 |
| rs10860891 | 12 | 103014757 | A | C | 7.85199e-09 | -0.22 | 0.038 |
| rs111837807 | 6 | 31121232 | C | T | 7.92866e-13 | 0.29 | 0.040 |
| rs13050728 | 21 | 34615210 | C | T | 2.30622e-12 | -0.19 | 0.027 |
| rs2109069 | 19 | 4719443 | A | G | 1.46386e-21 | 0.26 | 0.027 |
| rs35081325 | 3 | 45889921 | T | A | 5.28567e-46 | 0.63 | 0.044 |
